# Rational strategy for power doubling of monolithic multijunction III-V photovoltaics by accommodating attachable scattering waveguides

**DOI:** 10.1038/s41377-024-01628-6

**Published:** 2024-09-20

**Authors:** Shin Hyung Lee, Hyo Jin Kim, Jae-Hyun Kim, Gwang Yeol Park, Sun-Kyung Kim, Sung-Min Lee

**Affiliations:** 1 School of Electrical Engineering, Korea Advanced Institute of Science and Technology (KAIST), Daejeon, 34141 Republic of Korea; 2 Optoelectronic Convergence Research Center, Korea Photonics Technology Institute, Gwangju, 61007 Republic of Korea; 3 Department of Applied Physics, Kyung Hee University, Yongin, 17104 Republic of Korea; 4 Department of Electrical Engineering, Hanyang University, Seoul, 04763 Republic of Korea; 5 Division of Materials Science and Engineering, Hanyang University, Seoul, 04763 Republic of Korea

**Keywords:** Solar energy and photovoltaic technology, Electronics, photonics and device physics

## Abstract

While waveguide-based light concentrators offer significant advantages, their application has not been considered an interesting option for assisting multijunction or other two-terminal tandem solar cells. In this study, we present a simple yet effective approach to enhancing the output power of transfer-printed multijunction InGaP/GaAs solar cells. By utilizing a simply combinable waveguide concentrator featuring a coplanar waveguide with BaSO_4_ Mie scattering elements, we enable the simultaneous absorption of directly illuminated solar flux and indirectly waveguided flux. The deployment of cells is optimized for front-surface photon collection in monofacial cells. Through systematic comparisons across various waveguide parameters, supported by both experimental and theoretical quantifications, we demonstrate a remarkable improvement in the maximum output power of a 26%-efficient cell, achieving an enhancement of ~93% with the integration of the optimal scattering waveguide. Additionally, a series of supplementary tests are conducted to explore the effective waveguide size, validate enhancements in arrayed cell module performance, and assess the drawbacks associated with rear illumination. These findings provide a comprehensive understanding of our proposed approach towards advancing multi-junction photovoltaics.

## Abstract (web summary)

The maximum output power of transfer-printed multijunction InGaP/GaAs solar cells is enhanced by approximately 93% through cost-effective integration with a coplanar waveguide that includes BaSO_4_ Mie scattering elements.

## Graphical abstract

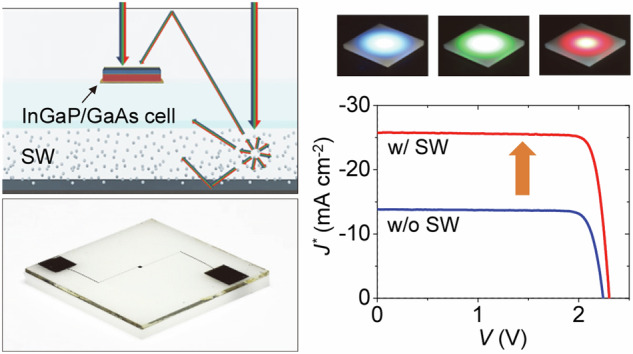

## Introduction

Monolithic multijunction III-V compound semiconductor solar cells are widely recognized as ultrahigh-performance photovoltaics, stemming from their favorable material properties such as direct bandgap, high carrier mobility, low-temperature coefficient, and superior radiative hardness^1–5^. These types of solar cells are simply configured into two-terminal photovoltaics without an increase in parasitic absorption loss, which is a practically attractive feature as the complexity of photovoltaic module implementation can be reduced^6,7^. Despite such compelling features, their high manufacturing cost has been a hurdle for the broad application in terrestrial photovoltaics^8^. One direct approach for improving their cost-effectiveness might be reducing materials consumption. This case can be achieved by designing minimal epitaxial stacks (e.g., lattice-matched structures) or reusing growth wafers (e.g., epitaxial lift-off process)^9–11^. Alternatively, an indirect approach to addressing this issue involves improving cell utilization by increasing power generation capability with light concentrators^12–14^. A concentrator intensifies the solar photon flux incident to solar cells, thus enabling solar cells to generate the boosted output power beyond a value available under unconcentrated illumination. However, the typical light concentrators comprising optics components are not actually cost-effective because of the sophisticated module assembly level and the essential maintenance system. Moreover, they do not operate properly under diffuse sunlight, impeding their effectiveness in reducing the levelized cost of electricity.

In contrast, waveguide-based light concentrators do not diminish the cost-reduction benefits due to simple module construction. The waveguide concentrator consists of a transparent multimode slab waveguide containing luminescent, scattering, or both elements capable of redirecting photon propagation^15–22^. By diverting photons towards solar cells embedded within the waveguide or mounted at the waveguide edges through total internal reflection, photon concentration becomes possible without the need for precise module construction. Its effective operation under diffusely incident sunlight is another notable advantage of the waveguide concentrator. Despite these advantages, the waveguide concentrator has not been considered a promising option for reinforcing multijunction or other two-terminal tandem solar cells^6,23,24^. This is due to several concerns regarding performance synergy. While the multijunction cells, typically designed as monofacial cells, require front-side photon incidence to achieve a balanced output current from series-connected subcells, the bifacial cell is an effective configuration to capture photons traveling within the waveguide. Additionally, as multijunction cells are designed to operate efficiently across the entire solar spectrum, the waveguide concentrator with spectral elements responding to a narrow photon spectrum (e.g., luminophores) may not be effective with them. Rigorous tuning of spectral elements in the waveguide concentrator can alleviate the ineffectiveness of their finite spectrum responses^6^; however, the level of output power amplification still remains limited, thus necessitating the development of more advanced waveguide concentrator technology compatible with high photovoltaic-capable multijunction cells.

In this regard, the present study introduces a straightforward yet effective approach to amplifying the output power of multijunction III-V solar cells by employing a simply combinable waveguide concentrator. Microscale InGaP/GaAs double-junction solar cells are arranged within a transparent slab medium through the transfer-printing assembly method, where the cell deployment is optimized to enhance front-surface photon collection, both for directly incident solar flux and waveguided solar flux. To enable broad-spectrum solar photons to be waveguided^20–22^, we separately prepare a photon scattering medium of a sticky elastomer slab with BaSO_4_ particles and attach it underneath the cell-incorporated transparent waveguide slab. The key features of the present approach are (i) the optimal integration design of multijunction cells with waveguides to concentrate broadband photon flux preferentially on the front cell surfaces and (ii) the simplicity of module implementation that allows considerable augmentation of the multijunction cell output power without the need for complicated process addition. Based on experimental characterization and numerical modeling, systematic investigations to access photon waveguide and collection properties at various waveguide parameters and scattering element conditions are conducted to find the maximum benefits of waveguide concentrators with multijunction III-V solar cells.

## Results

### Module design and fabrication

**Fig. 1:**
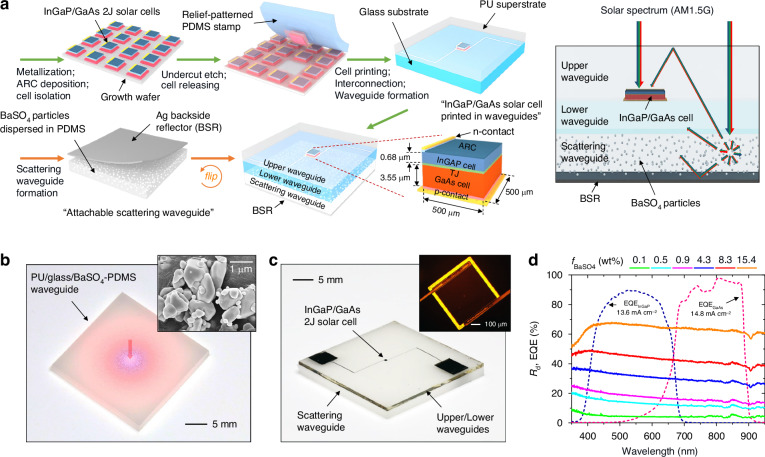
Module design and fabrication for InGaP/GaAs solar cell with scattering waveguide. **a** Schematic illustrations of summarized fabrication steps for the proposed module and their optical process under the solar spectrum. **b** Photographic image of the PU/glass/BaSO_4_-PDMS scattering waveguide under centrally incident red light. The inset indicates a scanning electron microscope (SEM) image of BaSO_4_ particles. **c** Photographic image of the integrated photovoltaic module. The inset depicts an optical micrograph of the transfer-printed InGaP/GaAs solar cell. **d** Measured *R*_d_ of the BaSO_4_-PDMS scattering layer at various *f*_BaSO4_s and measured EQE spectra of the InGaP/GaAs solar cell

Figure 1a schematically depicts the proposed module design and its fabrication steps. The module comprises two-terminal microscale InGaP/GaAs solar cells (area; *A*_c_ = 500 × 500 μm^2^) and upper (polyurethane; PU), lower (glass), and scattering (polydimethylsiloxane; PDMS) waveguide sublayers. The solar cells are positioned horizontally between the upper and lower waveguide sublayers. The scattering waveguide sublayer contains disorderly distributed BaSO_4_ particles, and an Ag backside reflector (BSR) is placed at the bottom surface of this waveguide. This module configuration allows two distinct routes for the incident solar photons to reach the solar cells. One route involves the direct photon incidence facilitated by the coplanar cell-to-waveguide arrangement. The other route follows an indirect path, in which the solar photons incident to the outside of the cells are redirected through collisions with the BaSO_4_ particles in the scattering waveguide sublayer, ultimately moving toward the embedded solar cells. This indirect route is well compatible with multijunction cell features: (i) A waveguide channel of the upper sublayer facilitates photon collection at the front cell surface. (ii) The scattering response across the broad spectral range empowers balanced photocurrent enhancements for the top and bottom subcells.

The module was created through straightforward fabrication processes. Initially, a package of releasable InGaP/GaAs solar cells was formed on a growth wafer, involving sequent steps such as contact metallization, antireflection coating (ARC, 60 nm ZnS), cell isolation, and undercut etching. Subsequently, a specific InGaP/GaAs cell was transfer-printed onto a glass substrate serving as a transparent lower waveguide sublayer (thickness; *t*_lw_ = 700 μm) using a relief-patterned PDMS stamp. Following the formation of interconnection electrodes and the addition of a transparent upper waveguide sublayer (PU, *t*_uw_ = 420 μm), a fully operational module with the InGaP/GaAs solar cell was ready. At this stage, the module, before incorporating the scattering waveguide sublayer, demonstrated a power conversion efficiency (PCE) of ~26.2% with a short-circuit current density (*J*_sc_) of ~13.8 mA cm^-2^, an open-circuit voltage (*V*_oc_) of ~2.24 V, and a fill-factor (FF) of ~84.7%. Detailed information on epitaxial stacks on the growth wafer and fabrication procedures can be found in the Methods section and Figs. S1 and S2. A scattering waveguide sublayer (*t*_sw_ = 1900 μm) was separately prepared by thermal curing of PDMS mixed with dispersed BaSO_4_ particles, where a fraction of BaSO_4_/PDMS in weight (*f*_BaSO4_) ranged from 0.05 to 15.4 wt%. An Ag BSR (*t* = 100 nm) was deposited afterward. Since this cured BaSO_4_-PDMS exhibited a self-adhesive property and hence easily adhered to a flat surface^25,26^, the final step for combining the scattering sublayer with the module was completed through a simple physical attachment process. A photographic image of the assembled upper/lower/scattering waveguide is presented in Fig. 1b, allowing for the observation of the spreading behavior of centrally incident photons within this waveguide. A complete InGaP/GaAs cell module integrated with the suggested waveguide combination is shown in Fig. 1c.

Balanced subcell photocurrents are crucial for enhancing the overall output current of InGaP/GaAs cells, given the series-connected subcell configuration. As depicted in the external quantum efficiency (EQE) response in Fig. 1d, the experimental sample without scattering elements, which represents the case of only direct photon incidence, exhibited reasonably balanced yet slightly discrepant photocurrent densities (*J*_ph_s) between the top and bottom subcells (*J*_ph,top_ = 13.6 mA cm^-2^ < *J*_ph,bot_ = 14.8 mA cm^-2^) under the non-concentrated AM1.5 G solar illumination. Therefore, photon flux indirectly supplied to the InGaP/GaAs cell from the waveguide needs to boost both subcell photocurrents, with a slight bias toward the current-limiting subcell (i.e., the top subcell) for optimal balance. The BaSO_4_ particles (size = 200 ~ 700 nm) dispersed in the PDMS waveguide exhibited the white scattering behavior that covered the entire absorption spectra of the top and bottom subcells (Figs. S3 and S4). This implies that scattered photons in the waveguide can influence both subcell photocurrents. Meanwhile, the overall diffuse reflectance (*R*_d_) of the BaSO_4_-PDMS scattering waveguide monotonically increased with an increase in the *f*_BaSO4_ due to raised photon collision events (Fig. 1d). Notably, *R*_d_ was moderately higher at shorter wavelengths, suggesting that scattered photons can contribute more effectively to the top subcell. The inclusion of an Ag BSR further amplified the *R*_d_ of the BaSO_4_-PDMS, indicating that stronger scattering can be anticipated at a given *f*_BaSO4_ (Fig. S5).

### Waveguide parameter optimization

**Fig. 2:**
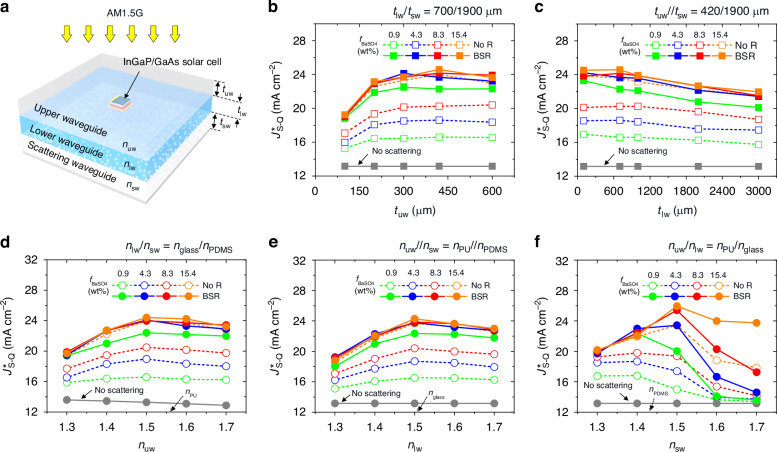
Numerical calculations for waveguide parameter optimization. **a** Schematic illustrations of the simulated module configuration. Calculated J^*^_S-Q_s for PU/glass/BaSO_4_-PDMS (*n*_PU_/*n*_glass_/*n*_PDMS_ ≈ 1.56/1.51/1.43) waveguide modules at various *t*_uw_s under fixed *t*_lw_/*t*_sw_ = 700/1900 μm (**b**) and at various *t*_lw_s under fixed *t*_uw_//*t*_sw_ = 420//1900 μm (**c**). Calculated J^*^_S-Q_s for PU/glass/BaSO_4_-PDMS (*t*_uw_/*t*_lw_/*t*_sw_ = 420/700/1900 μm) waveguide modules if *n*_uw_ (**d**), *n*_lw_ (**e**), and *n*_sw_ (**f**) change

The photon delivery property of the waveguide concentrator depends on several factors, including the geometric and optical parameters of the upper, lower, and scattering waveguide sublayers. These parameters affect the quantity and distribution of photons trapped and guided within the waveguide, ultimately regulating the amount of collected photons at the front surface of the InGaP/GaAs cell embedded within. To gain insights into the effects of these parameters and identify their optimal values for maximizing cell performance, we conducted optical modeling for the experimental module (as depicted in Fig. 2a) with various thicknesses (*t*_uw_, *t*_lw_, *t*_sw_) and refractive indices (*n*_uw_, *n*_lw_, *n*_sw_) of the upper, lower, and scattering waveguide sublayers. Using the 3-dimensional ray-tracing simulation, an amount of collected photon flux at the front cell surface was calculated, where details of the simulation setup and representative ray-tracing results can be found in Table. S1 and Fig. S6. Assuming that the collected photon flux obtained in the ray-tracing simulation was normally incident to an InGaP/GaAs cell, the photon absorption in the emitter and base layers, which are primarily responsible for photocurrent generation^6,27^, was assessed using the multilayer transfer-matrix formula^28,29^. A quantitative comparison of the cell performance was enabled by evaluating the Shockley-Queisser current limit (denoted as J^*^_S-Q_) based on the detailed balance analyses given by the equation below^30–34^.

1 $${J}_{{\rm{S}}-{\rm{Q}}}^{\ast }=q{\int }_{0}^{\infty }{A}_{{\rm{e}}/{\rm{b}}}(E){\varPhi }_{{\rm{S}}}(E)dE$$

Here, a superscript ‘*’ indicates the cell area (*A*_c_) normalization. *q*, *A*_e/b,_ Φ_s_, *E* are the electron charge, the emitter/base absorptance, the collected solar flux at the front cell surface, and the photon energy, respectively. The output J^*^_S-Q_ of the InGaP/GaAs cell was determined by choosing a smaller J^*^_S-Q_ value against the InGaP and GaAs subcells, considering the current matching rule of series-connected two-terminal multijunction configuration^33^.

Figure 2b, c present the calculated J^*^_S-Q_s for the experimental upper/lower/scattering configuration of PU/glass/BaSO_4_-PDMS (*n*_PU_/*n*_glass_/*n*_PDMS_ ≈ 1.56/1.51/1.43) when changing *t*_uw_ and *t*_lw_ values at different *f*_BaSO4_ levels of the BaSO_4_-PDMS. Under fixed *t*_lw_ and *t*_sw_ (700 and 1900 μm), the J^*^_S-Q_ exhibited a specific trend when *t*_uw_ was increased from 100 to 600 μm (Fig. 2b). Initially, there was an increase in J^*^_S-Q_, followed by a saturation effect. This trend indicates that photon guidance to the front cell surface is facilitated by the appropriately thick upper sublayer serving the above-cell waveguide channel in the coplanar-type arrangement. Simultaneously, a higher *f*_BaSO4_ in the scattering sublayer resulted in a larger J^*^_S-Q_, regardless of *t*_uw_. This effect was due to more photons being scattered, contributing to the waveguiding process. The impact of increasing *f*_BaSO4_ on J^*^_S-Q_ varied depending on whether a BSR was included or not. With increasing *f*_BaSO4_, the module without the BSR (referred to as the BSR-less module) presented a gradual J^*^_S-Q_ improvement; by contrast, the module with the BSR (referred to as the BSR-added module) showed a rapid increase in J^*^_S-Q_ followed by saturation. This behavior can be explained by the augmented photon scattering in the BSR-added module, similar to the intensified scattering effect observed in the high-*f*_BaSO4_ case. Note that if *t*_uw_ exceeded a certain threshold (approximately over 400 μm), the J^*^_S-Q_ tended to decrease for modules with strong photon scattering (e.g., the BSR-less modules with *f*_BaSO4_ ≥ 15.4 wt%, the BSR-added modules with *f*_BaSO4_ ≥ 4.3 wt%) due to increased propagation loss. Further details on propagation loss will be discussed later. Meanwhile, increasing *t*_lw_ while keeping *t*_uw_ and *t*_sw_ constant (420 and 1900 μm) had a negative effect on J^*^_S-Q_ for all module cases (Fig. 2c). This is because the lower waveguide sublayer served as a pathway for photons that were not captured at the front cell surface. However, modules with *f*_BaSO4_ values exceeding 8.3 wt% showed relatively stable J^*^_S-Q_ until *t*_lw_ reached ~700 μm. This stability was attributed to less contribution of long-distance traveling photons to J^*^_S-Q_, which provided a design margin for *t*_lw_.

The J^*^_S-Q_ variations with changes in sublayer refractive indices are provided in Fig. 2d–f. In these calculations, one sublayer of the experimental PU/glass/BaSO_4_-PDMS waveguide (*t*_uw_/*t*_lw_/*t*_sw_ = 420/700/1900 μm) was replaced by a non-extinction material with a refractive index ranging from 1.3 to 1.7, while constraining other sublayer materials consistent. Figure 2d depicts the case of changing *n*_uw_ at fixed *n*_lw_/*n*_sw_ of *n*_glass_/*n*_PDMS_ (≈ 1.51/1.43). Regardless of *f*_BaSO4_ and BSR conditions, all modules showed their highest J^*^_S-Q_ values at *n*_uw_ around *n*_lw_, because suppressed reflection at an interface of the upper/lower sublayers facilitated the photon quantity at the above-cell waveguide channel. In contrast to the slight J^*^_S-Q_ reduction for the case of *n*_uw_ > *n*_lw_, the J^*^_S-Q_ reduction for the opposite case was significant. This accelerated J^*^_S-Q_ reduction can be understood as follows: when *n*_uw_ was below *n*_sw_ (a material where momentum change occurs), a condition for total internal reflection could be satisfied, meaning that scattered photons with high in-plane momentum did not enter the upper sublayer. In Fig. 2e, if fixing *n*_uw_//*n*_sw_ to *n*_PU_//*n*_PDMS_ (≈ 1.56//1.43), the maximum J^*^_S-Q_ for all modules appeared at *n*_lw_ around 1.5, which is in the middle of *n*_uw_ and *n*_sw_. This result is expected, as this *n*_lw_ condition matched the minimum reflection loss for scattered photons toward the above-waveguide channel, considering both the reflection boundaries between scattering/lower sublayers and lower/upper sublayers. At *n*_lw_ < *n*_sw_, a critically low J^*^_S-Q_ was observed due to total internal reflection, similar to the case of *n*_uw_ < *n*_sw_ in Fig. 2d. Meantime, a case of changing *n*_sw_ under fixed *n*_uw_/*n*_lw_ (*n*_PU_/*n*_glass_ ≈ 1.56/1.51) in Fig. 2f exhibited J^*^_S-Q_ variation behavior corresponding to the cases of changing *n*_uw_ or *n*_lw_. The best J^*^_S-Q_ tended to exist at *n*_sw_ around *n*_lw_ due to minimal reflection loss, while the worst J^*^_S-Q_ emerged at *n*_sw_ > *n*_lw_ due to total internal reflection generation. However, at the condition of *n*_sw_ < *n*_lw_ (also *n*_sw_ < *n*_uw_), which was suboptimal for reflection loss, the highest J^*^_S-Q_ was unexpectedly observed for modules with less photon scattering (e.g., the BSR-less modules with *f*_BaSO4_ ≤ 8.3 wt%, the BSR-added modules with *f*_BaSO4_ ≤ 0.9 wt%). This J^*^_S-Q_ variation behavior was because the weak scattering sublayer provided a comparatively active propagation path acting as a photon loss channel, and decreasing *n*_sw_ alleviated the propagating photon density in this loss channel.

### Photovoltaic performance

**Fig. 3:**
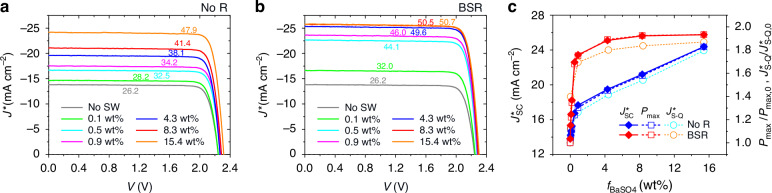
Photovoltaic performance of InGaP/GaAs solar cell with scattering waveguide. Representative J^*^-*V* curves of the BSR-less (**a**) and BSR-added (**b**) modules measured under the AM 1.5 G solar spectrum (100 mW cm^–2^) at various *f*_BaSO4_s. The term ‘no SW’ means the control module without BaSO_4_ particles. **c** Measured J^*^ and *P*_max_/*P*_max,0_ and calculated J^*^_S-Q_/J^*^_S-Q,0_ for the modules as a function of *f*_BaSO4_. *P*_max,0_ and J^*^_S-Q,0_ indicate *P*_max_ and J^*^_S-Q_ of the control module, respectively

Figure 3a, b shows representative density-voltage (J^*^-*V*) curves of experiment InGaP/GaAs modules without and with BSR, respectively, at various *f*_BaSO4_ levels under the stimulated AM 1.5 G solar spectrum (100 mW cm^–2^). Derived photovoltaic parameters of these modules are provided in Table 1. Compared to the control module without the BaSO_4_ scattering elements (i.e., PU/glass/PDMS waveguide) (J^*^_sc_ = 13.8 mA cm^-2^, *V*_oc_ = 2.24 V, FF = 84.7%, *P*_max_/*A*_c_ = 26.2 mW cm^–2^), the modules incorporating the BaSO_4_-PDMS scattering sublayer dramatically increased the J^*^_sc_ and slightly enhanced the *V*_oc_ without degradation of the FF, meaning that boosting power generation by the scattering sublayer was operated well. When examining the case of the BSR-less modules (Fig. 3a), we observe that the J^*^_sc_ rose from 13.8 to 17.66 mA cm^–2^ even at a low *f*_BaSO4_ level, such as *f*_BaSO4_ = 0.9 wt%. The J^*^_sc_ nearly linearly increased further as promoting the *f*_BaSO4_ from 0.9 wt%, resulting in 24.38 mA cm^–2^ and hence *P*_max_/*A*_c_ = 47.9 mW cm^–2^ at *f*_BaSO4_ = 15.4 wt% (82.6% power increase against the control device value). By contrast, in the case of the BSR-added modules (Fig. 3b), the J^*^_sc_ augmented rapidly as increasing *f*_BaSO4_ level initially and became saturated after *f*_BaSO4_ ≥ 4.3 wt%. The calculated J^*^_S-Q_s (Fig. S7) values were compared with the measured J^*^_sc_s, as shown in Fig. 3c. The consistent variation with changes in *f*_BaSO4_ was observed, indicating analyses implemented in the calculation process are valid for the measured results: The photon scattering contribution is enhanced more at higher *f*_BaSO4_ and adding the BSR. The best module performance can be found in the BSR-added module with *f*_BaSO4_ = 15.4 wt%, which was J^*^_sc_ = 25.75 mA cm^–2^, *V*_oc_ = ~2.30 V, FF = ~85.5%, and *P*_max_/*A*_c_ = 50.68 mW cm^–2^ (93.2% power increase against the control device value). Note that minor discrepancies between the calculated and measured values would come from the assumptions during calculation for simplicity (e.g., the normal incidence of waveguided photons, photocurrent generation only at emitter/base). Meanwhile, it is noteworthy that no performance degradation was observed at the module with the scattering waveguide, as it efficiently operates under obliquely incident light (Fig. S8). The temperature coefficient of this module was measured to -0.275% °C^-1^, reflecting a slight increase in *J*_sc_ and substantial decreases in *V*_oc_ and FF (Fig. S9), as expected from the literature^35^. The sudden drop in FF at high temperatures (> 140°C) was most likely due to an increase in series resistance caused by the thermal expansion of via-holes used for metal interconnection.

**Table 1:** Photovoltaic characteristics of various conditions for InGaP/GaAs solar cell under the AM 1.5 G illumination (100 mW cm^-2^)

*f*_BaSO4_ (wt%)	J^*^_sc_ (mA cm^-2^)		*V*_oc_ (V)		FF (%)		*P*_max_/*A*_c_ (mW cm^-2^)^a^	
	No R	BSR	No R	BSR	No R	BSR	No R	BSR
**0 (control)**	13.80 ± 0.04^b^		2.24 ± 0.02		84.7 ± 0.7		26.23 ± 0.30	
**0.1**	14.68 ± 0.03	16.61 ± 0.01	2.24 ± 0.01	2.25 ± 0.01	85.5 ± 0.2	85.5 ± 0.2	28.18 ± 0.07	32.01 ± 0.08
**0.5**	16.74 ± 0.10	22.60 ± 0.07	2.26 ± 0.01	2.27 ± 0.01	85.7 ± 0.2	85.6 ± 0.2	32.50 ± 0.20	44.05 ± 0.05
**0.9**	17.66 ± 0.20	23.44 ± 0.25	2.26 ± 0.02	2.29 ± 0.02	85.5 ± 0.5	85.5 ± 0.2	34.20 ± 0.46	45.95 ± 0.50
**4.3**	19.48 ± 0.29	25.10 ± 0.43	2.28 ± 0.02	2.30 ± 0.03	85.6 ± 0.3	85.6 ± 0.3	38.07 ± 0.40	49.56 ± 0.50
**8.3**	21.20 ± 0.44	25.62 ± 0.43	2.28 ± 0.02	2.30 ± 0.03	85.6 ± 0.3	85.6 ± 0.2	41.43 ± 0.59	50.48 ± 1.07
**15.4**	24.38 ± 0.21	25.75 ± 0.21	2.29 ± 0.03	2.30 ± 0.03	85.6 ± 0.2	85.5 ± 0.3	47.90 ± 0.15	50.68 ± 0.37

^a^*P*_max_/*A*_c_ indicates the maximum output power normalized by a cell area

^b^These are statistical values of average and standard deviation obtained from 10 measurements

### Photon waveguide properties

**Fig. 4:**
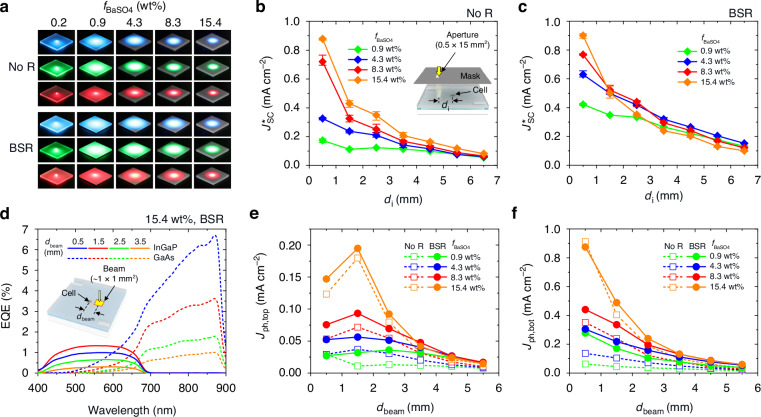
Photon delivery properties of scattering waveguide module. **a** Photographic images of the PU/glass/BaSO_4_-PDMS waveguides without and with BSR under centrally incident blue, green, and red lights at various *f*_BaSO4_s. **b,** **c**, Measured J^*^_sc_s of the BSR-less (**b**) and BSR-added (**c**) modules under the area-confined (0.5 × 15 mm^2^ bar) AM 1.5 G solar spectrum as a function of *d*_i_, center-to-edge distance between cell and illumination aperture, at various *f*_BaSO4_s. **d**, Measured EQE spectra of the 15.4 wt% BSR-added module at various distances (*d*_beam_) between the cell center and beam edge. Estimated *J*_ph,top_ (**e**) and *J*_ph,bot_ (**f**) from the EQE spectra for the BSR-less and BSR-added modules as increasing *d*_beam_ at various *f*_BaSO4_s

To evaluate the photon delivery performance of the experimental PU/glass/BaSO_4_-PDMS waveguide, series tests were conducted at various *f*_BaSO4_ values and conditions of the BSR inclusion. Figure 4a provides photographic images of the waveguide samples, depicting light-spreading for centrally incident point sources of blue, green, or red lights. Regardless of the color, light-spreading due to photon scattering rapidly decreased as *f*_BaSO4_ increased. This implies that the photon scattering contribution of high-*f*_BaSO4_ waveguides is spatially limited to nearby cells. When comparing the waveguide samples without and with BSR, extended light-spreading was found in the BSR samples under an identical *f*_BaSO4_ condition. This is because the BSR could create a double path for downward photons, which compensates for the limited light scattering. This behavior can be illustrated in the experiment of the area-confined (0.5 mm width and 15 mm length) AM 1.5 G solar illumination distant from the cell using a black mask (center-to-edge gap between cell and illumination aperture; *d*_i_). As described in Fig. 4b (BSR-less module) and 4c (BSR-added module), the measured J^*^_sc_s of all modules exponentially decayed along *d*_i_ and their decay level intensified with raising *f*_BaSO4_. This is due to the exponential extinction of propagating photons, especially in dense scattering components. Quantitatively, the decay rate (α) of the BSR-less module increased from 0.33 to 0.42 to 0.55 mm^-1^ as *f*_BaSO4_ changed from 4.3 to 8.3 to 15.4 wt%. On the other hand, the decay rate of the BSR-added module was relatively alleviated at the same *f*_BaSO4_ conditions (α change from 0.13 to 0.33 to 0.54 mm^–1^), as expected in the light-spreading configuration test of Fig. 4a. It needs to be mentioned that if only the BaSO_4_-PDMS scattering sublayer exists without the support of transparent PU/glass sublayers, the photon waveguide deteriorates more rapidly at high *f*_BaSO4_, as depicted in Fig. S10.

While the experiments of Fig. 4b, c showed the highest J^*^_sc_ at the smallest *d*_i_ of 0.5 mm, the scattered photons in immediate proximity to the cell cannot be effectively captured on the front cell surface because enough space is not prepared for redirecting photons by the scattering events behind the cell to the above-cell waveguide. The calculation results for situations of Fig. 4b, c demonstrate this nature, as shown in Fig. S11. Since we believe the accuracy of the narrow bar aperture alignment could not be fully secured in the experiment, we implemented another test to identify the distant illumination effect using a comparative point beam (~1 × 1 mm^2^) of the EQE measurement system. Figures 4d and S12 provide measured EQE spectra for the InGaP top cell and the GaAs bottom cell at various distances between the cell and EQE beam (gap between cell center and beam edge; *d*_beam_). One obvious fact is that the scattered photons preferentially move to the back surface of the cell as the scattering events occur behind the cell (i.e., top-junction-limited cells). Therefore, designs for higher photon density in the above-cell waveguide channel are essential. The photocurrents of the InGaP (*J*_ph,top_) and GaAs (*J*_ph,bot_) cells derived from the measured EQE spectra are summarized in Fig. 4e, f. As mentioned above, the *J*_ph,top_ exponentially decreased as *d*_beam_ increased; however, the maximum *J*_ph,top_ did not appear at the smallest *d*_beam_ due to insufficient space for guiding the scattered photons to the front cell surface. In contrast, the *J*_ph,bot_ presents its maximum value at the smallest *d*_beam_, which is recognizable because there is no need for circumventive movement of the scattered photons.

### Module characterizations at various conditions

**Fig. 5:**
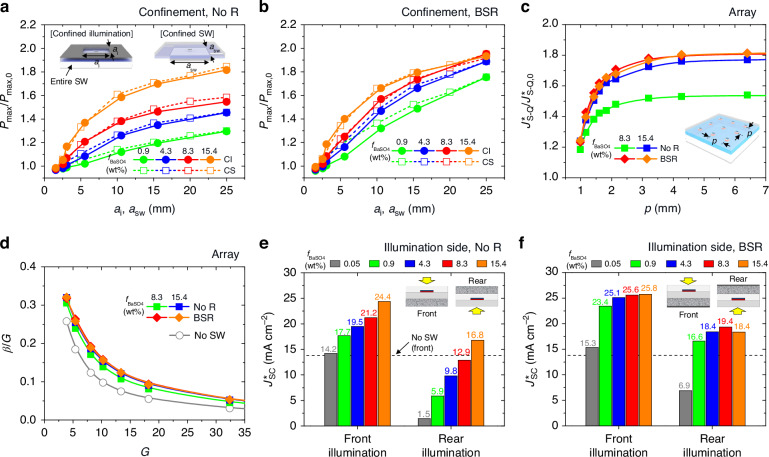
Module properties at various conditions. Measured power gain *P*_max_/*P*_max,0_ of the BSR-less (**a**) and BSR-added (**b**) modules for spatially square-confined cell illumination (side length: *a*_i_) or BaSO_4_-PDMS scattering sublayer (side length: *a*_sw_) at various *f*_BaSO4_s. **c** Calculated current gain J^*^_S-Q_/J^*^_S-Q,0_ of the 8.3 and 15.4 wt% arrayed cell modules without and with BSR as a function of cell period *p*. **d** Derived *β*/*G* factors for modules of (**c**) as a function of geometry gain *G*. *β*/*G* of the control module (no SW) also appears for comparison. Measured J^*^_sc_s of the BSR-less (**e**) and BSR-added (**f**) modules when the BaSO_4_-PDMS scattering sublayer exists on rear (front illumination) or front (rear illumination) surfaces at various *f*_BaSO4_s

To further characterize the scattering waveguide concentrator modules, we implemented several tests, as provided in Fig. 5. Firstly, the effective size of the BaSO_4_-PDMS scattering sublayer was examined with the setup of (i) spatially confined illumination (square aperture side length; *a*_i_) on the centrally positioned cell and (ii) spatially confined scattering sublayer (square side length; *a*_sw_) under the AM 1.5 solar spectrum. Figure 5a, b presents the measured power gain of *P*_max_/*P*_max,0_ (where *P*_max,0_ indicates *P*_max_ of the control device) as *a*_i_ or *a*_sw_ increased for the BSR-less and BSR-added modules, respectively. While gradually saturating, *P*_max_/*P*_max,0_ of all cases was monotonically raised as *a*_i_ or *a*_sw_ was enlarged. This implies that even though the propagation decay is strict in the scattering waveguide, a larger waveguide can produce a higher *P*_max_/*P*_max,0_ enhancement effect. At similar *a*_i_ and *a*_sw_ values, *P*_max_/*P*_max,0_ with an identical *f*_BaSO4_ showed analogous levels, indicating that the influence of the scattering sublayer confinement was comparable to that of the light confinement. As expected, a promptly increased *P*_max_/*P*_max,0_ along *a*_i_ (or *a*_sw_) and its higher maximum were observed in the BSR-added module due to the BSR effect on expanding photon propagation and scattering. The consistent results of this confinement test can be found in the calculation given in Fig. S13.

Secondly, the effective cell period in the waveguide was investigated for modules consisting of a rectangular cell array. Since our calculations deeply correspond to the experiments, this test was conducted only with the calculation for various cell periods (*p*). Figure 5c provides the current gain of J^*^_S-Q_/J^*^_S-Q,0_ (where J^*^_S-Q,0_ represents the J^*^_S-Q_ of the control device) for the best-performing modules at various *p* values. While the J^*^_S-Q_/J^*^_S-Q,0_ of all modules rapidly increased as *p* expanded initially, it saturated shortly at *p* > ~1.8 mm. It is noteworthy that although a wider illumination may lead to a larger power gain in the confined illumination test, it cannot be understood as forbidding neighboring cells nearby because a certain amount of waveguided photons bypass the neighboring cells. Thus, such a dense array (*p* = 1.8 ~ 3.2 mm) of cells could achieve the full benefit from the scattering waveguide. To more explicitly assess the waveguide module performance, we employed the parameters of the current gain, *β* = J^*^_S-Q_/J^*^_S-Q,0_, and the geometry gain, *G* = *p*^2^/*a*_cell_^2^, which derived a factor, *β*/*G* = *J*_S-Q_/J^*^_S-Q,0_, a ratio of *J*_S-Q_ of the arrayed cell module against J^*^_S-Q,0_ of the single control cell^6,36^. Assuming the waveguide cost is negligible compared to the cell cost, the *G* factor indicates the cost reduction ratio and the *β*/*G* factor represents the PCE reduction ratio for the arrayed cell module against the single cell^6,37,38^. As illustrated in Fig. 5d, the decrease in *β*/*G* level along with the increase in *G* was considerably mitigated with the scattering waveguide, implying the arrayed cell module can attain the PCE improvement effect by simply incorporating the BaSO_4_-PDMS sublayer. For instance, when a relatively large *G* of 13.4, the *β*/*G* factor was improved from 0.075 to 0.124 (65.5% higher) by introducing the scattering waveguide.

Thirdly, we changed the position of the BaSO_4_-PDMS scattering sublayer from bottom to top and switched the illumination direction accordingly. This test shows the front illumination case is better for obtaining the high J^*^_sc_, as the real illumination case can generate the J^*^_sc_ only with the scattered photons behind. Figure 5e, f provides the results of this test for BSR-less and BSR-added modules, respectively. In the case of the BSR-less modules, the J^*^_sc_ produced by the rear illumination gradually increased as *f*_BaSO4_ increased, eventually exceeding J^*^_sc,0_ when *f*_BaSO4_ = 15.4 wt% (J^*^_sc_ = 16.8 mA cm^–2^) (Fig. 5e). This increase in J^*^_sc_ for the rear illumination was over 1.5 times compared to that for the front illumination. When the BSR was added, the scattering and waveguide performance improved, resulting in a further increase in J^*^_sc_ up to 19.4 mA cm^-2^. However, while the real illumination case can lead to a higher net increase of J^*^_sc_, the front illumination case presents a higher absolute J^*^_sc_ due to the contribution of direct solar photon incidence.

## Discussion

In summary, we proposed a simple strategy to double the output power of multijunction III-V solar cells and systematically studied its principles and properties. We developed a module consisting of microscale InGaP/GaAs transfer-printed coplanarly between PU and glass, with BaSO_4_ scattering particles-dispersed PDMS attached behind. By regulating the geometrical and optical parameters of the waveguide sublayers to simultaneously collect the flux of directly incident solar photons and waveguided scattered photons at the front surface of the embedded solar cell, we achieved approximately 93% enhancement in output power compared to the module before incorporating the BaSO_4_-dispersed scattering waveguide. Through a series of additional tests, we examined the effective waveguide size, the validity of arrayed cell module performance enhancement, and the disadvantage of the rear illumination case.

This study demonstrated a straightforward route to improve the power generation of multijunction solar modules comprised of mini-cell arrays. As the multijunction cells should adopt the monofacial configuration, typical BSR or related diffuse BSR cannot operate well without sophisticated but uncomplicated waveguide designs. While the micro-lens technique may offer superior light concentration, the present scattering waveguide technique does not require as strict a sun-tracking system as the micro-lens concentrators due to its focal-free operation. The scattering waveguide could potentially complement the micro-lens concentrator, particularly in scenarios involving off-normal light incidence. We anticipate our design process and characterization results will provide versatile solution options for enhancing multijunction solar cells.

## Materials and methods

### Fabrication of transfer-printed InGaP/GaAs solar cells

Epitaxial materials of InGaP/GaAs double-junction solar cells were grown on a p-type (001) GaAs wafer using metal-organic chemical vapor deposition (MOCVD, AIX 200/4). The fabrication of transfer-printed double-junction solar microcells started with the electron beam evaporation (Infovion) of n-type ohmic metal contact (AuGe/Ni/Au = 100/30/100 nm), followed by wet chemical etching of n^+^-GaAs top contact layer using a mixture of citric acid and hydrogen peroxide (C_6_H_8_O_7_:H_2_O_2_ = 4:1 by volume). Subsequently, the formation of cell mesa (500 × 500 μm^2^) structure by photolithography (AZ5214, AZ Electronic Materials) and wet chemical etching in a mixture of 15 ml HBr, 1 g K_2_Cr_2_O_7_ and 15 ml deionized (DI) water were conducted. After the thermal deposition of ZnS on the exposed window layer as a single-layer ARC, an additional mesa area (560 × 560 μm^2^) was defined to isolate individual cells by wet chemical etching (HBr/ K_2_Cr_2_O_7_/DI water) and the exposed AlAs was partially etched by HF. Photoresist (AZ4620, AZ Electronic Materials) was then spin-coated as a polymeric anchor, followed by the formation of etch holes on the second mesa region by photolithography and wet chemical etching (HBr/K_2_Cr_2_O_7_/DI). Transferable cells were released from the growth wafer with a PDMS elastomeric stamp (Sylgard 184, Dow Corning) after the selective undercut etching of the AlAs sacrificial layer in diluted HCl solution (HCl:DI water = 3:1 by volume). Released microcells were printed on a glass substrate using photocurable adhesive (~1 μm), and thereafter the remaining p-GaAs base and p-In_0.51_Ga_0.49_P back surface field (BSF) layer on the additional mesa were removed in C_6_H_8_O_7_/H_2_O_2_ (4:1 by volume) and H_3_PO_4_/HCl/DI water (1:1:1 by volume), respectively. On the exposed p-GaAs bottom contact layer, the p-type ohmic metal contact (Cr/Au = 20/100 nm) was then deposited by electron beam evaporation, and electrical passivation except for via-hole of p- and n- contacts was then configured using an insulating layer (SU-8 2002, AZ Electronic Materials). Lastly, the metal bus electrodes (Cr/Au/Cr = 30/1500/30 nm) were deposited for interconnecting metal contacts by thermal evaporation, and transparent PU (NOA61, Norland Products) were drop-casted and subsequently cured to form a top-side waveguide.

### Fabrication of attachable scattering waveguide sublayer

To prepare a detachable scattering waveguide sublayer, the weighed BaSO4 (97.5%) powder was mixed with PDMS polymer and curing agent (10:1 by weight). The mixture was degassed and then transferred into glass square molds. After thermally curing at 65 °C for 120 min, the thickness of the resulting scattering waveguide was 1.9 mm. The scattering waveguide defined in the same size as the glass substrate was attached at the back of the glass substrate. For adding the BSR, a thermally deposited Ag mirror was placed at the rear surface of the scattering waveguide.

### Characterization of optical and photovoltaic properties

Diffuse transmission and reflection of scattering waveguides were measured by a homemade optical setup consisting of a white light source, spectrometer (Maya pro 2000, Ocean Optics), and integrating sphere (RTC-060-SF, Labsphere). Current-voltage curves of InGaP/GaAs solar cells were obtained at room temperature using a source meter (Series 2400, Keithley) and a full-spectrum solar simulator with AM 1.5 G spectrum filter (K3000, McScience), where the 1-sun intensity was calibrated using a reference cell (K801S-K070, McScience). EQE measurements of solar cells were conducted by a commercial quantum efficiency measurement system (QuantX 300, Oriel) equipped with a white bias source (IQE-LIGHT-BIAS, Oriel).

### Ray-optic simulation

Optical simulations of modules were carried out using commercial ray-tracing software (LightTools, Synopsys). To improve the accuracy (an error rate of ~5%), the number of incident rays of the light source for each wavelength (ranging from 400 to 900 nm) was set to 10^7^. Some of the refractive indices of the optical modeling were designed based on the software database, and the others were taken from the literature^38–41^. To simulate the scattering waveguide similar to the experiment, the BaSO_4_ powder dispersed in PDMS was set to Mie scattering particles with a size of 10–700 nm, and the measured diffuse reflection of scattering waveguides was used to calculate the number density of BaSO_4_ particles. Details of the simulation setup can be found in Table. S1.

## Supplementary information

Supplementary Information
