# Supplementary material for: Rational strategy for power doubling of monolithic multijunction III-V photovoltaics by accommodating attachable scattering waveguides
Source: Light Sci Appl. 2024 Sep 20;13:261. doi: 10.1038/s41377-024-01628-6 (PMC11413176; doi:10.1038/s41377-024-01628-6)
Supplement: Supplementary file 1 — Supplementary Information [file 41377_2024_1628_MOESM1_ESM.pdf]

Supplementary Information for

Rational strategy for power doubling of monolithic multijunction III-V photovoltaics by accommodating attachable scattering waveguides

Shin Hyung Lee,[+] Hyo Jin Kim,[+] Jae-Hyun Kim, Gwang Yeol Park, Sun-Kyung Kim, and Sung-Min Lee[*]

Table of Contents

1. Supplementary Figures and Legends S-2

Figs. S1 ~ S13

2. Supplementary Tables S-11

Table. S1

3. Supplementary References S-12

1. Supplementary Figures and Legends

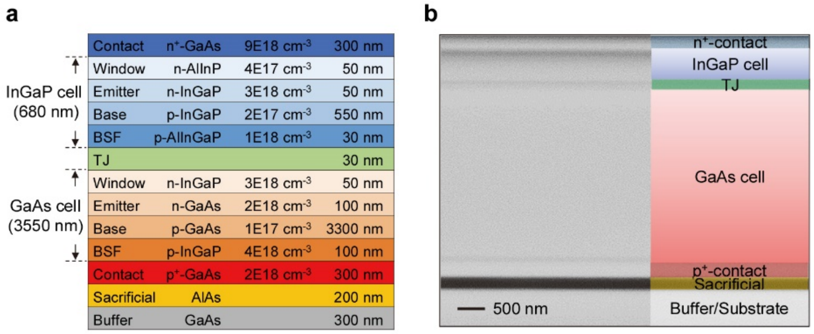

Fig. S1 Epitaxial stacks of InGaP/GaAs solar cells. **a**, Schematic structure of releasable InGaP/GaAs solar cell stacks epitaxially grown on a GaAs growth wafer, along with specifications of each layer, including materials, doping concentrations, and thicknesses. **b**, Cross-sectional scanning electron microscope (SEM) images of epitaxial stacks.

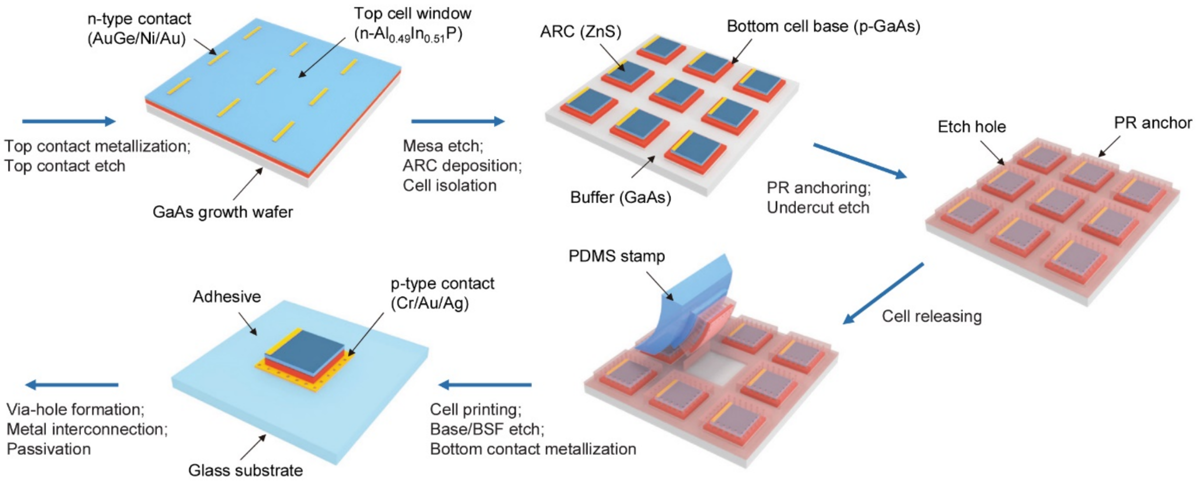

Fig. S2 Process flow of InGaP/GaAs solar cells. Schematic illustration of processing steps from epitaxial stacks on the growth wafer to a complete cell on a glass substrate.

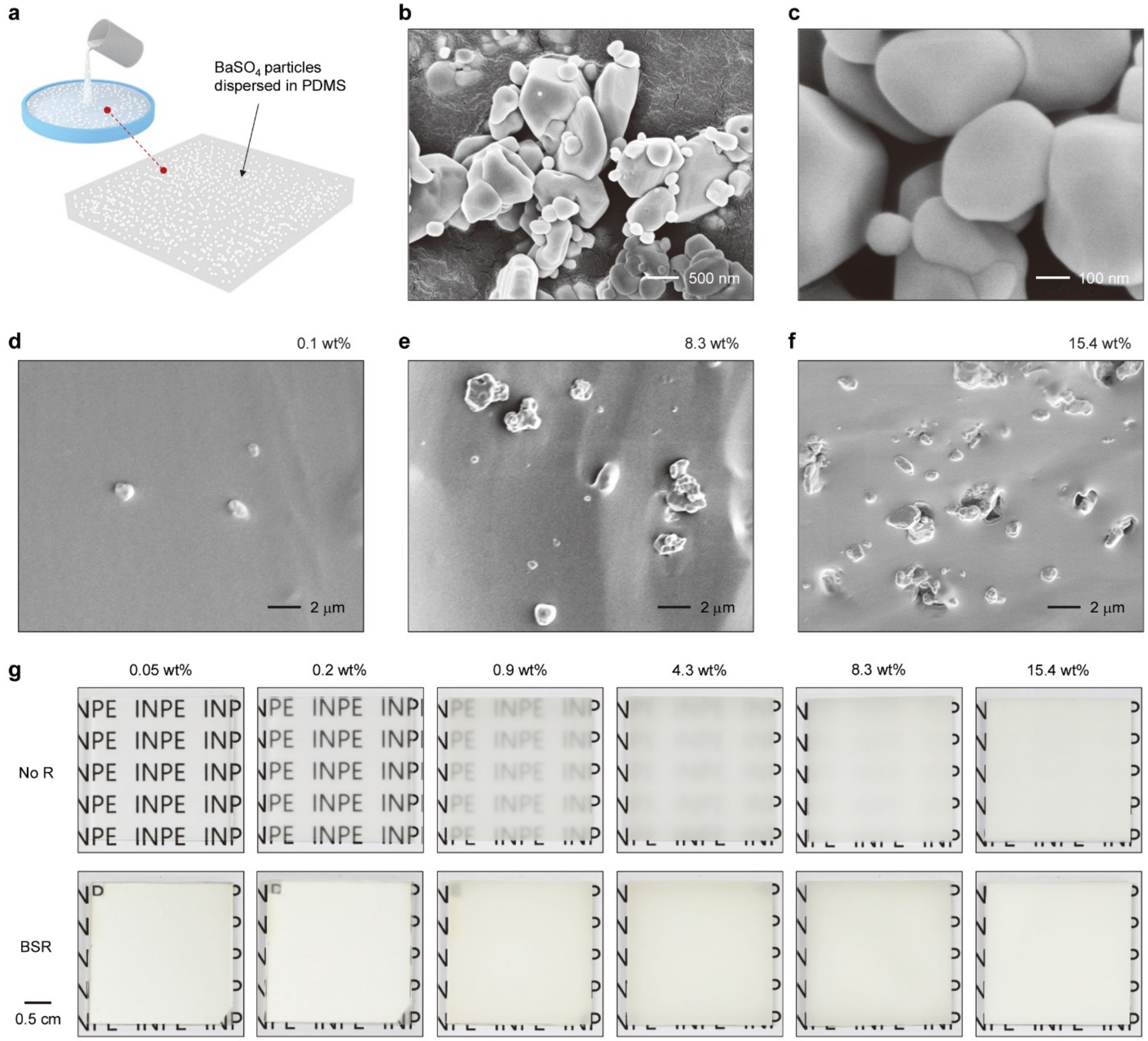

Fig. S3 White scattering behavior of $BaSO_4$-PDMS scattering waveguide. **a**, Schematic illustration of dispersing $BaSO_4$ particles into PDMS and the fabricated result. **b**,**c**, Low-magnification (**b**) and high-magnification (**c**) SEM images of the $BaSO_4$ particles. **d**-**f**, Top-view SEM images of $BaSO_4$-PDMS scattering waveguide with f_{BaSO4}s of 0.1 (**d**), 8.3 (**e**), and 15.4 wt% (**f**). **g**, Photograph of $BaSO_4$-PDMS scattering waveguides without and with BSR at various f_{BaSO4}s.

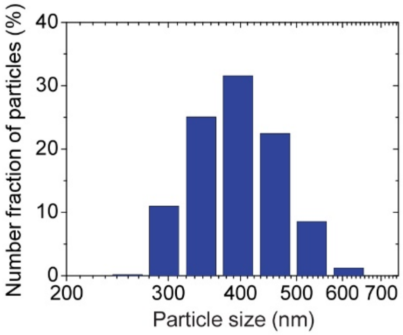

Fig. S4 $BaSO_4$ particle size distribution. Measured size distribution of $BaSO_4$ particles using the dynamic light scattering system. The particles were dispersed in isopropyl alcohol.

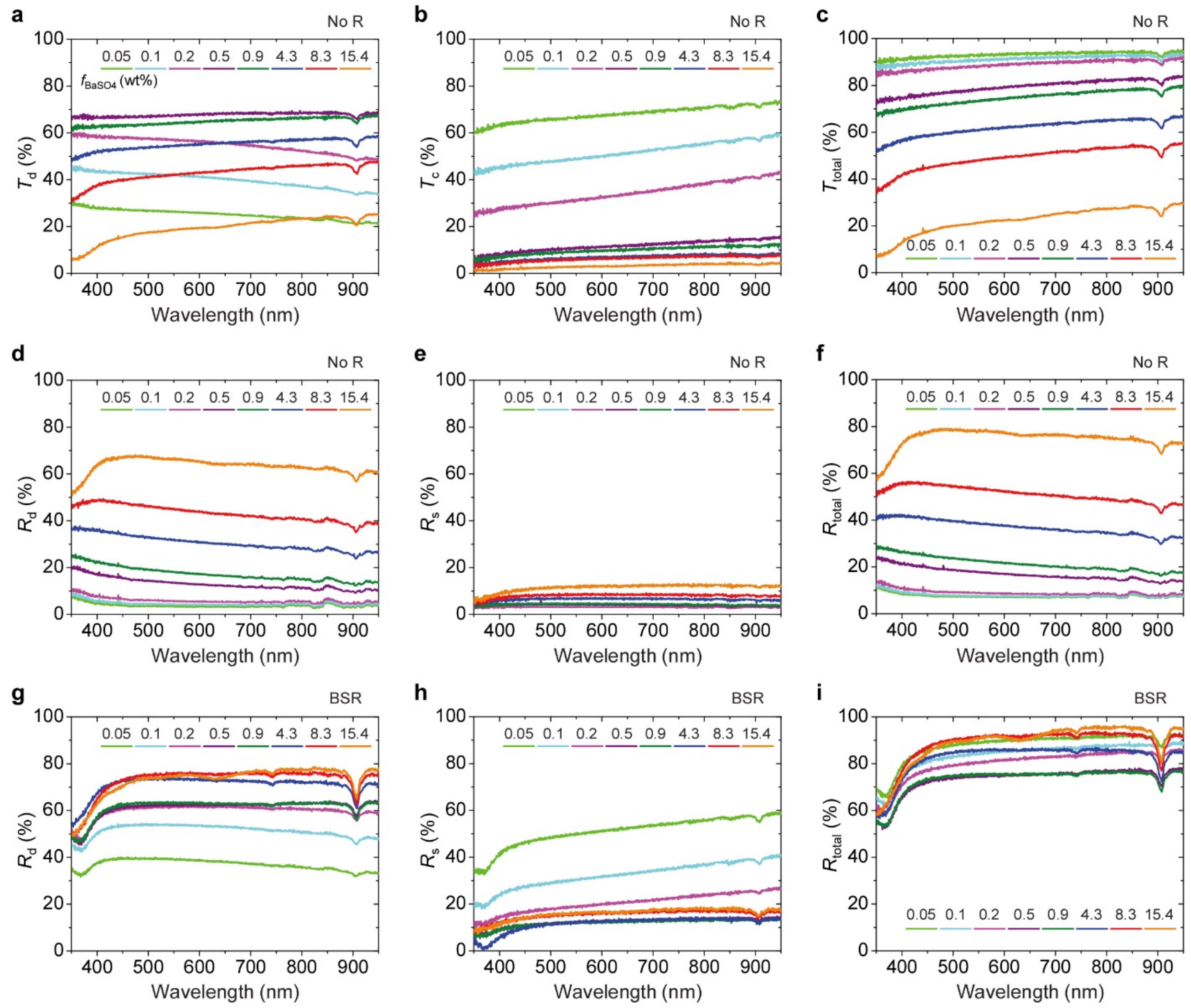

Fig. S5 Optical properties of the scattering waveguide. **a**-**c**, Measured spectra of diffuse (T_d) (**a**), collimated (T_c) (**b**), and total ($T_{total} = T_d + T_c$) (**c**) transmittance for scattering waveguides without BSR at various f_{BaSO4}s. **d**-**f**, Measured spectra of diffuse (R_d) (**d**), specular (R_s) (**e**), and total ($R_{total} = R_d + R_s$) (**f**) reflectance for the samples corresponding to (**a**-**c**). **g**-**i**, Measured R_d (**g**), R_s (**h**), and R_{total} (**i**) reflectance for scattering waveguides with BSR at various f_{BaSO4}s.

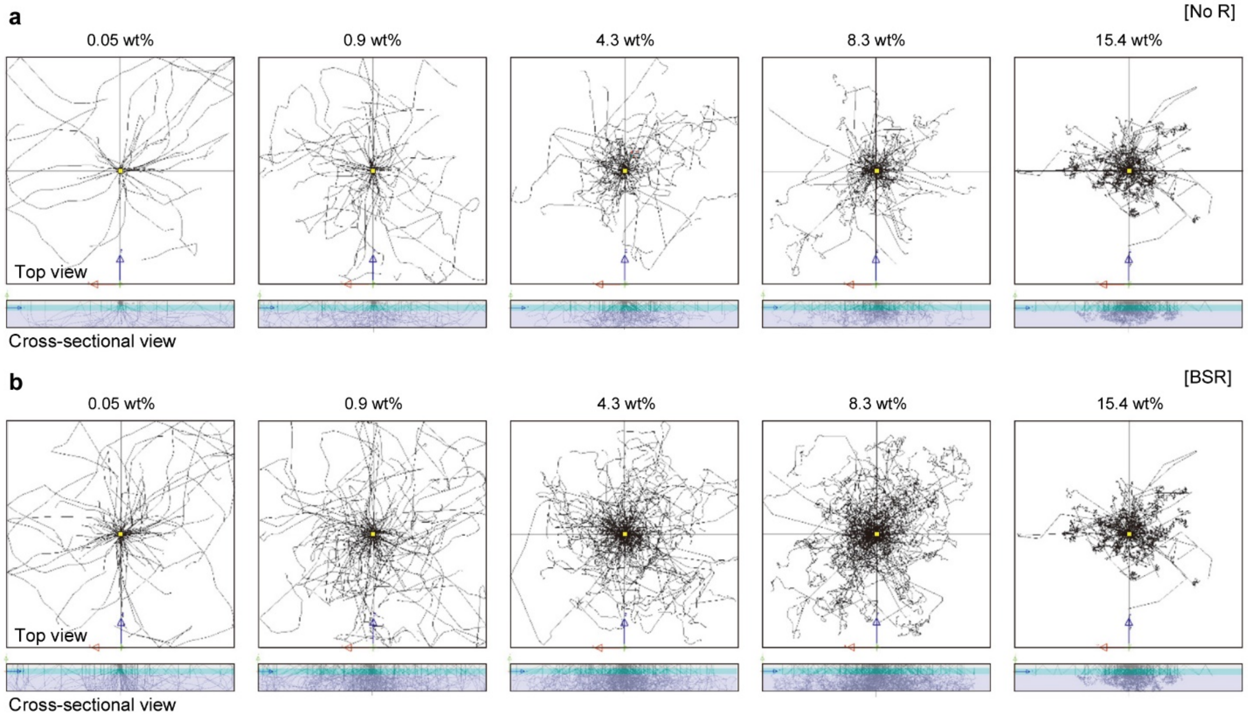

Fig. S6 Ray-tracing diagrams of scattering waveguides. **a**,**b**, Top- and side-view of the ray tracing (λ = 600 nm) guided into the front cell surface for the BSR-less (**a**) and BSR-added (**b**) modules at various f_{BaSO4}s.

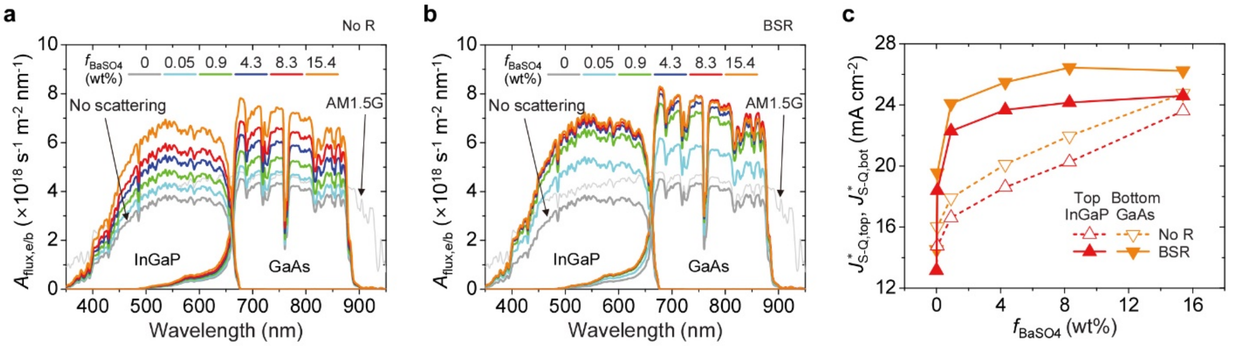

Fig. S7 Optical modeling of various scattering waveguide modules with a single InGaP/ GaAs solar cell. **a**,**b**, Calculated absorbed photon flux in emitter/base ($A_{flux,e/b}$) of each subcell for BSR-less (**a**) and BSR-added (**b**) modules at various f_{BaSO4}s. The light gray line indicates the AM 1.5G solar flux. **c**, Calculated J^*_{S-Q}s of top ($J^*_{S-Q,top}$) and bottom ($J^*_{S-Q,bot}$) subcells for modules corresponding to (**a**,**b**)

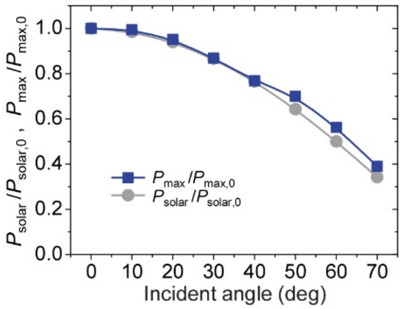

Fig. S8 Angular dependence of the InGaP/GaAs module performance with scattering waveguide. Measured P_{max} of the 15.4 wt% module normalized by $P_{max,0}$ (P_{max} under the normally illuminated AM 1.5G solar spectrum) at various incident angles. $P_{solar}/P_{solar,0}$ indicates a ratio of incident solar power, which is yielded as $P_{solar}/P_{solar,0} = \cos\theta$.

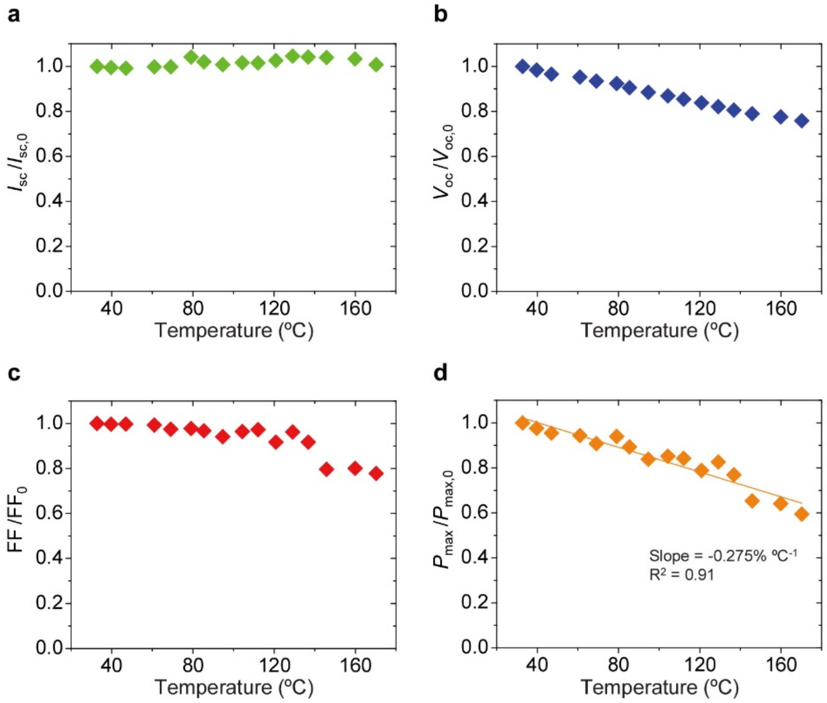

Fig. S9 Temperature dependence of the InGaP/GaAs module performance with scattering waveguide. Measured I_{sc} (**a**), V_{oc} (**b**), FF (**c**), and P_{max} (**d**) of the 15.4 wt% module normalized by their initial values ($I_{sc,0}$, $V_{oc,0}$, FF$_0$, and $P_{max,0}$ at 32.8ºC), respectively, as a function of the sample temperature.

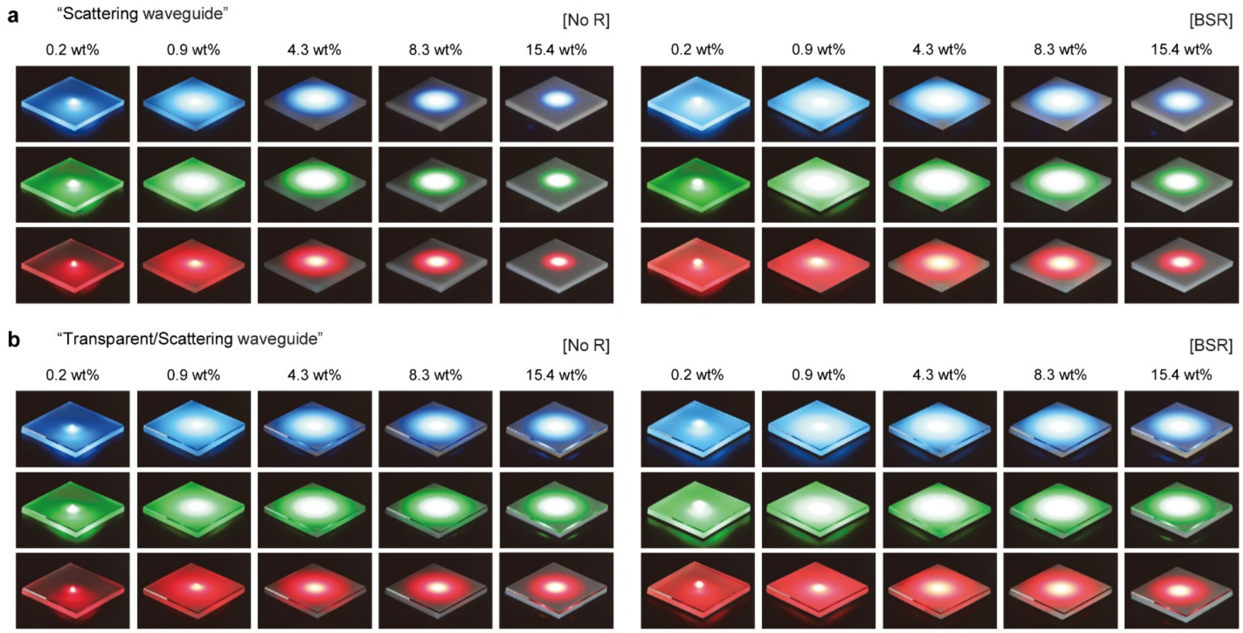

Fig. S10 Photon waveguide behavior. **a**,**b**, Photographic images of the $BaSO_4$-PDMS scattering waveguide (**a**) and the PU/glass/$BaSO_4$-PDMS scattering waveguide (**b**) under centrally incident blue (465 nm), green (530 nm), and red (630 nm) lights at various f_{BaSO4}s.

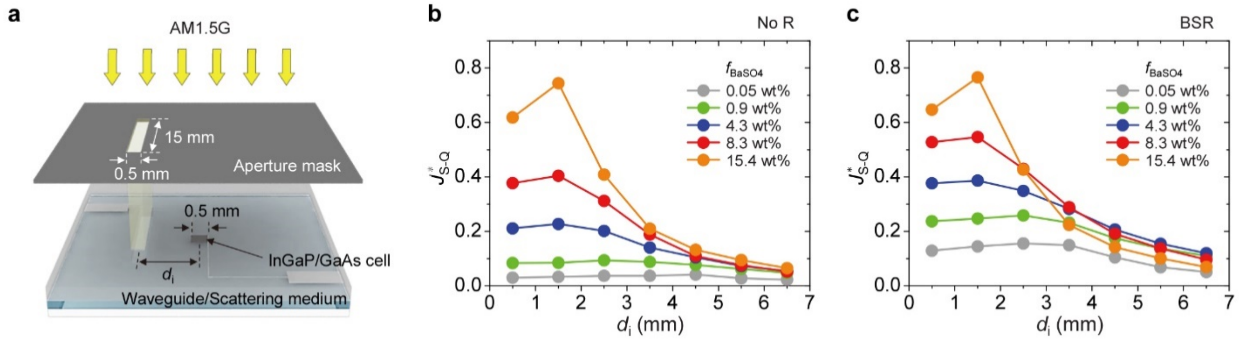

Fig. S11 Photon delivery calculation. **a**, Schematic illustrations of scattering waveguide modules for the photon delivery study. **b**,**c**, Calculated J^*_{S-Q}s of the BSR-less (**b**) and BSR-added (**c**) modules under the area-confined (0.5 × 15 mm^2 bar) AM 1.5G solar spectrum as a function of d_i, center-to-edge distance between cell and illumination aperture, at various f_{BaSO4}s

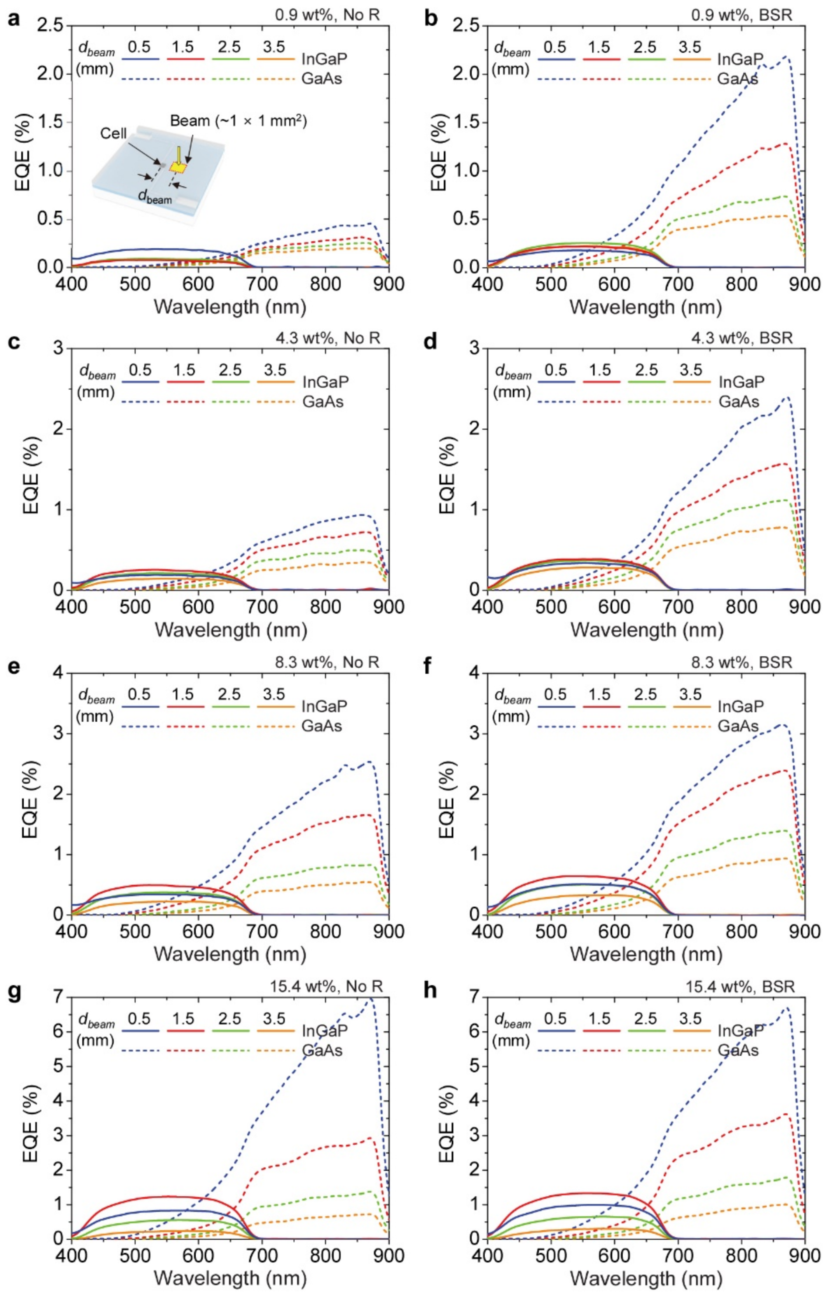

Fig. S12 EQE measurement under distant incident beams. a-h, Measured EQE spectra of the BSR-less (**a**,**c**,**e**,**g**) and BSR-added (**b**,**d**,**f**,**h**) module at various distances (d_{beam}) between the cell center and beam edge. f_{BaSO4} values are 0.9 (**a**,**b**), 4,3 (**c**,**d**), 8.3 (**e**,**f**), and 15.4 wt% (**g**,**h**), respectively.

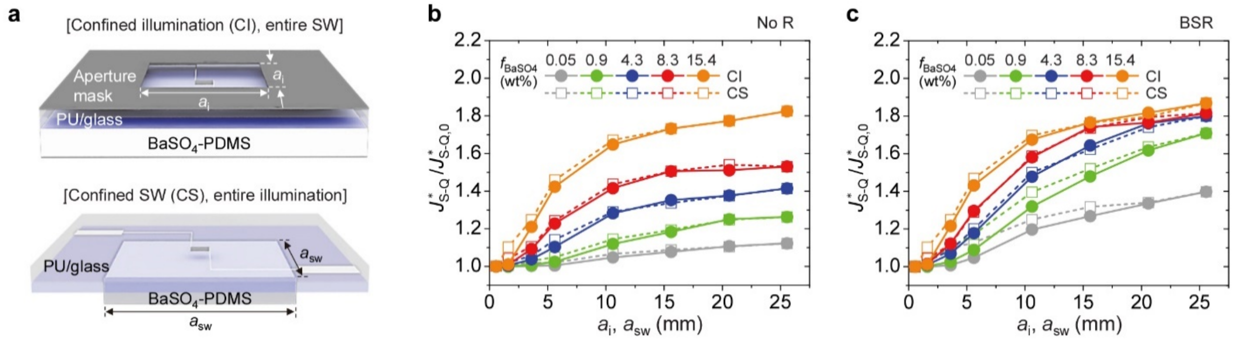

Fig. S13 Confinement effect calculation. **a**, Schematic illustration of the setup for spatially square-confined cell illumination (side length: a_i) or $BaSO_4$-PDMS scattering sublayer (side length: a_{sw}). **b**,**c**, Calculated current gain $J^*_{S\text{-}Q}/J^*_{S\text{-}Q,0}$ of the BSR-less (**b**) and BSR-added (**c**) modules as a function of a_i or a_{sw} at various f_{BaSO4}s.

2. Supplementary Tables

Table. S1 3-dimensional ray-tracing simulation setup for calcuating the photon flux incident on the front cell surface.

Default setting of ray-tracing simulation

Material type	Homogeneous
Optical properties	Smooth optical (Fresnel loss)
Number of rays	10,000,000
Error prediction	< 5 %

Refractive indices of materials

Wavelength	400 nm	500 nm	600 nm	700 nm	800 nm	900 nm
n_{PU}[1]	1.581	1.567	1.559	1.554	1.55	1.547
n_{sub}[2]	1.525	1.515	1.509	1.506	1.504	1.502
n_{PDMS}[3]	1.448	1.436	1.43	1.426	1.424	1.422
n_{Ag}[4]	0.05	0.05	0.055	0.041	0.037	0.04
k_{Ag}[4]	2.104	3.131	4.01	4.803	5.57	6.371
n_{gaas}[5]	4.374	4.308	3.92	3.773	3.684	3.666
k_{gaas}[5]	2.145	0.427	0.231	0.14	0.086	0.08

Properties of scattering waveguide as particle density

Weight percentage of particles	15.4 wt%	8.3 wt%	4.3 wt%	0.9 wt%
Particle size distribution analyzing	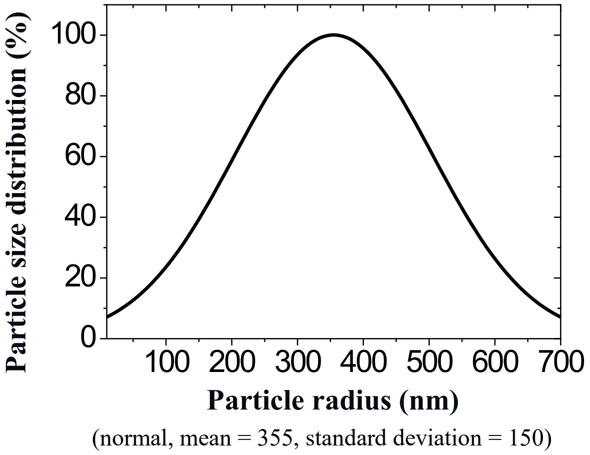 (normal, mean = 355, standard deviation = 150)			
Particle density (mm^{-3})	181,100,000	32,260,000	13,070,000	4,725,000
Mean free path (mm)	0.0083404	0.046821	0.11557	0.31967

3. Supplementary References

1. Norland optical adhesive 61. https://www.norlandprod.com/adhesives/noa61pg2.html (2021).
2. Eagle XG AMLCD glass. https://valleydesign.com/Datasheets/Corning-Eagle-XG-glass.pdf (2006).
3. Schneider, F., Draheim, J., Kamberger, R. & Wallrabe, U. Process and material properties of polydimethylsiloxane (PDMS) for Optical MEMS. *Sensors and Actuators A: Physical* 151, 95-99 (2009).
4. Johnson, P. B. & Christy, R.-W. Optical constants of the noble metals. *Physical Review B* **6**, 4370 (1972).
5. Aspnes, D. E., Kelso, S. M., Logan, R. A. & Bhat, R. Optical properties of Al x Ga1− x As. *Journal of Applied Physics* 60, 754-767 (1986).
